# Supplementary material for: Organ-Specific Glucose Uptake: Does Sex Matter?
Source: Cells. 2022 Jul 16;11(14):2217. doi: 10.3390/cells11142217 (PMC9323353; doi:10.3390/cells11142217)
Supplement: Supplementary file 1 [file cells-11-02217-s001.zip › cells-1768752-supplementary.pdf]

Supplementary Materials:

Table S1. Body weights of mice before injection of FDG and PET scan

Female Chow	Male Chow	Female HFD	Male HFD
17.8	23.9	18.88	32.7
17.3	25.7	22.20	28.65
18.2	22.2	18.89	30.67
16.8	23	29.04	32.7
17.5	25.9	21.2	31.14
17.6	23.5	20.24	28.33
17.4	23.4	17.82	25.8
16.8	25	19.94	23.28
17.6	28.8	16.54	25.45
18.8	26.3	18.56	30.61
14.6	27.4		30.65
20.3	25.7		
17.8	25.1		
17.3	18.9		
18.2			
16.8			

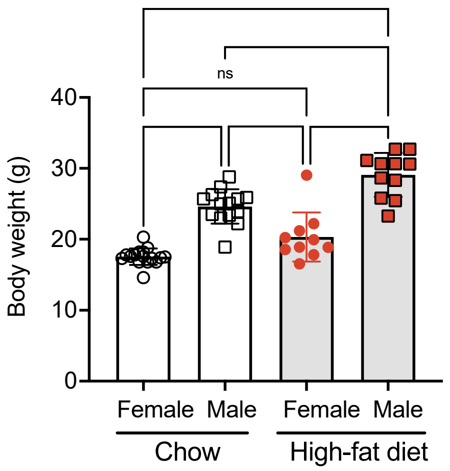

Figure S1. Bar graphs showing average body weights of female and male mice on chow and high-fat diets taken on the day just before injection of FDG and PET scanning from data in Table S1. Female mice do not gain weight on HFD in two weeks, whereas male mice do. ANOVA, Sidak's multiple comparison. *n*=10-16

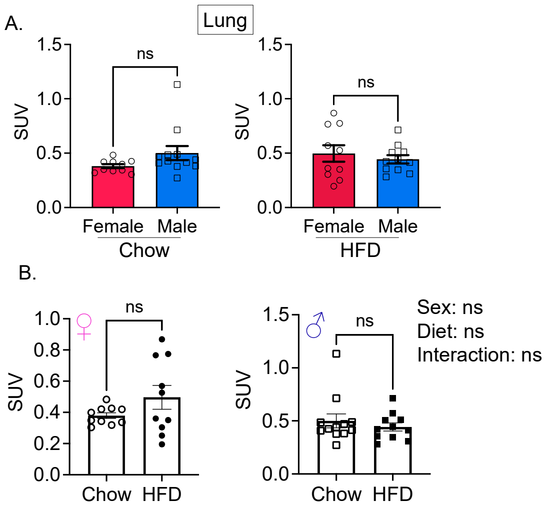

Figure S2. Sex and Diet do not influence glucose uptake in the lung. (**A**) No sex difference was noted in FDG uptake in lungs of young male and female mice. (**B**) High-fat diet did not significantly alter FDG uptake in lungs of male and female mice. *n*=8-9/sex. Two-way ANOVA was performed followed by Mann-Whitney *post hoc* analysis. Circles: female; squares: male.

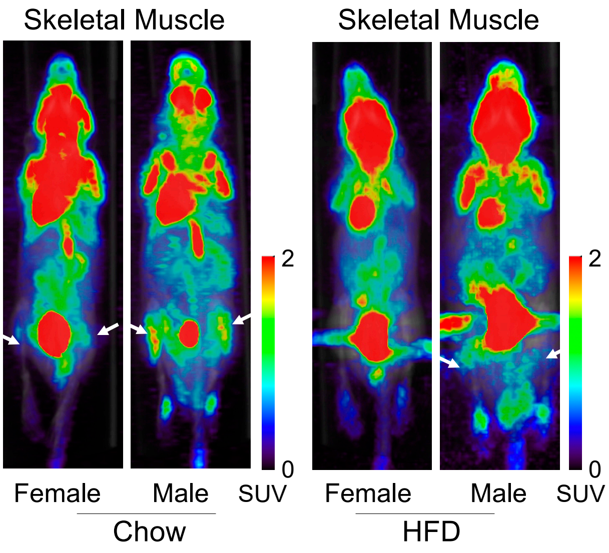

Figure S3. High-fat diet (HFD) decreased FDG uptake in the skeletal muscle. Representative PET scans from male and female mice are shown to illustrate volume of interest (VOI) measurements in skeletal muscle for FDG uptake (arrows).
